# Understanding the profile of community health workers in breast cancer screening education: women’s preferences and insights from a qualitative focus group study

**DOI:** 10.1186/s12939-025-02508-0

**Published:** 2025-07-04

**Authors:** Ilka Jacobs, Dorien Vanden Bossche, Sara Willems, Katrien Vanthomme

**Affiliations:** 1 Unit Epidemiology of Chronic Diseases, Department of Public Health and Primary Care, Ghent University, Ghent, Belgium; 2 Unit Family Medicine, Department of Public Health and Primary Care, Ghent University, Ghent, Belgium; 3 Unit Equity in (Primary) Health Care, Department of Public Health and Primary Care, Ghent University, Ghent, Belgium

**Keywords:** Community health workers, Breast cancer screening, Qualitative content analysis, Focus group interviews, Equity in healthcare

## Abstract

**Background:**

Despite efforts to reduce disparities in breast cancer screening uptake in Flanders, certain population groups, such as women with a lower income, a lower educational attainment, women living further from screening units, and with a migration background, remain less likely to participate in the population-based mammography screening program. Community Health Workers (CHWs) are effectively involved in breast cancer screening programs in various countries to reach underserved women. In Flanders, Belgium, the involvement of CHWs in breast cancer screening programs is relatively recent. However, little is known about the preferences of the target population regarding the potential profile of a CHW in breast cancer screening education in Flanders. This study aims to explore this gap.

**Methods:**

Four focus group discussions were conducted between August and December 2023. Data were analysed using Qualitative Content Analysis to identify key categories, which were subsequently organized according to Kok et al.’s framework of CHW performance.

**Results:**

The results emphasize the importance of the desired competencies of CHWs, including professional knowledge and expertise, personal experience in shaping competencies, effective communication skills, cultural awareness and motivational and supportive competencies. Empathy, sociability, and adaptability were identified as key attitudes for fostering trust and creating supportive relationships. Trust emerged as a central theme, where female CHWs were preferred for their ability to enhance safety and trust, especially when they had prior experience with breast cancer screening.

**Conclusion:**

The findings of this study provide actionable insights regarding the profile of CHWs involved in breast cancer screening education, underscoring the need for a balanced combination of attributes to address the specific preferences of the target population. By integrating the target populations’ perspectives, this research bridges gaps in existing CHW performance frameworks, offering a more comprehensive understanding of CHW performance. Recruiting CHWs with the right combination of competencies, interpersonal skills and attitudes is critical for the success of breast cancer education programs. Further research is needed to explore these findings in other contexts.

**Supplementary Information:**

The online version contains supplementary material available at 10.1186/s12939-025-02508-0.

## Background

Cancer represents a significant societal, public health, and economic challenge, with an estimated 20.0 million new cases and 9.7 million cancer deaths worldwide in 2022 [1]. Female breast cancer ranks as the second most frequently diagnosed cancer, accounting for 11.6% of all total cancer cases. In women, it is the most commonly diagnosed cancer and the leading cause of cancer death worldwide, representing nearly one-quarter of cancer cases and one-sixth of cancer-related deaths in women [1]. In Belgium, breast cancer remains a significant health concern, with 11,192 newly diagnosed cases in 2022 [2]. In Flanders, the Northern region in Belgium, which comprises 58% of the Belgian population [3], the age-standardized incidence rate in 2022 was 185.8 new cases per 100,000 women [2]. In contrast, the most recent mortality data are from 2021, when breast cancer resulted in 2,048 deaths in Belgium, corresponding to 29.7 deaths per 100,000 women in Flanders [2]. To reduce breast cancer mortality, many countries, including Belgium, have implemented population-based mammography screening programs (PMSP). In Belgium, healthcare policies such as PMSPs fall under the jurisdiction of the regions. The Flemish Region introduced its own PMSP in June 2001, targeting women aged 50–69 years in accordance with international recommendations from the World Health Organization (WHO) [4] and the European Commission Initiative on Breast Cancer - Guidelines Development Group [5]. The management, coordination, and supervision of the Flemish PMSP is carried out by the Centre for Cancer Detection (CCD), in collaboration with the Belgian Cancer Registry. Eligible women receive a biennial invitation from the CCD for screening, which involves a two-view mammogram of both breasts. No ultrasound or clinical breast examination is included in this procedure [6]. The Flemish PMSP has demonstrated a 51% decrease in breast cancer-specific mortality rates, based on outcomes recorded up to 31 December 2017, compared to opportunistic screening or no screening [7]. However, adequate participation at population level is a crucial component for the efficacy of a PMSP [8]. Despite specific efforts of the Flemish PMSP to reach all women, notable disparities in participation in the PMSP still exist. Low-income women, women with lower educational attainment, woman living further from screening units, and women having a migration background, are less likely to participate in the PMSP [8, 9].

Increasingly, research supports the importance of “proportional universalism” as a strategy to address health inequity [10], ensuring that interventions are universal -available to the entire population- but also proportionate to the level of need. This means that while everyone benefits from the intervention, the intensity and scale of support are tailored to address the specific needs of different groups, with a focus on those who are most disadvantaged [11]. In many countries, community health workers (CHWs) play a crucial role in preventive healthcare by embodying the principle of proportionate universalism to reach individuals who might otherwise be missed by broad, universal approaches. Yet, despite the widespread involvement of CHWs in numerous health initiatives, there remains limited consensus on their precise role, profile, and the different names or titles by which they are referred to [12]. According to the WHO, CHWs are *‘community members who are selected by their communities*,* accountable to them*,* supported by the health system*,* yet not necessarily integrated into its formal structure*,* and who typically have shorter training than professional healthcare workers’* [13]. The United States (U.S). Department of Health and Human Services offers a more comprehensive definition, describing CHWs as ‘*lay community members*,* often sharing ethnicity*,* language*,* socioeconomic status and life experiences with those they serve. They work*,* either for pay or as volunteers*,* with the local healthcare system in both urban and rural settings*,* offering services such as health education*,* interpretation*,* advocacy*,* and assist people in receiving the care they need’* [14]. CHWs go by various titles, including ‘community health advisors’, ‘lay health trainers’, ‘lay health workers’, ‘lay health advocates’, ‘outreach workers’, and ‘peer health educators’ [12, 14]. Due to their deep understanding of the communities they serve, CHWs are uniquely positioned to address specific health issues, such as cancer screening, to community members. In many countries, particularly in the U.S., there is a long tradition of involving CHWs in breast cancer screening initiatives to address disparities in access to care and participation in screening programs [15–26]. Their familiarity with the community enables them to build trust and effectively navigate social norms and barriers, thereby increasing the likelihood that screening interventions are tailored to the community’s needs [16, 17]. CHWs play a crucial role in these initiatives by educating individuals about breast cancer and screening procedures, guiding them through the healthcare system, and promoting preventive health behaviours through direct demonstration [15, 19]. In some interventions they visit women at home, while in others they provide support in community-based educational sessions [17, 23]. CHWs are typically community leaders or peer educators who possess relevant life experiences, making them relatable to the populations they serve [18, 20]. They are predominantly female, often bilingual, and may have backgrounds that enhance their understanding of the cultural contexts of the communities they work in [23, 25]. As navigators, CHWs perform outreach for breast cancer screening through workshops and health fairs to recruit women for mammography [21, 24]. Additionally, they address barriers to screening by providing motivation and logistical support for healthcare visits [25, 26].

Although CHWs play a key role in addressing health disparities in breast cancer screening access and participation, there is limited evidence regarding their role and profile in the Flemish context. In Flanders, CHWs are increasingly being deployed to enhance access to and participation in breast cancer screening programs. To develop effective strategies for engaging CHWs in these programs, it is crucial to understand the preferences of the target population, being Flemish women who face barriers to participation in the PMSP. Therefore, this research aims to explore the preferences of women in Flanders who are less likely to participate in the PMSP, regarding the desired profile of a CHW within breast cancer screening education. With this information, suitable CHWs can be identified to meet the specific needs of women in Flanders.

## Methods

### Approach

A descriptive qualitative methodology utilizing focus group discussions (FGDs) was adopted to investigate the preferred profile of CHWs involved in breast cancer screening education in Flanders. During the FGDs, participants responded to open-ended questions regarding the ideal CHW profile, focusing on the qualities, skills, and traits necessary to effectively address the needs of the target population. The discussions involved women from socio-demographic groups less likely to engage in the PMSP [8, 9], with the aim of capturing their perspectives on a CHW profile that aligns with their cultural and community-specific contexts. To ensure the quality of this study, the consolidated criteria for reporting qualitative research [27] and the standards for reporting qualitative research checklist were followed [28], as outlined in [additional file 1].

### Setting

This study was conducted in the city of Ronse. This city has been chosen because (1) the total coverage rate for breast cancer screening (54,8%) is less than the average in Flanders (64.7%) [29]; (2) Ronse exhibits several factors linked to higher non-participation in PMSP, such as an ethnically diverse population and an average income per habitant below the national average [30]; (3) there are no ongoing interventions related to breast cancer screening; and (4) the concept of the CHW is not yet known in this city, which allows participants to talk about their preferences from an unbiased perspective. All organizations in the city’s region that work with women from groups who are less likely to participate in the PMSP were invited to recruit women for the study. Two organizations agreed to recruit participants.

### Sampling

Eligible women were contacted by employees from the two organisations using a purposive sampling strategy. These organisations were specifically asked to recruit women belonging to the target group, namely women aged 50–69 from groups who are less likely to participate in the PMSP. All women from this target group who expressed an interest in the study were eligible to participate, with non-eligible participants (e.g., those outside the 50–60 age range) excluded from analysis. Full open disclosures of study purpose, procedures, risks, and benefits were provided using a study leaflet available in Dutch, English and French. The sample size was based on data saturation [31] with an expected number of three to five FGDs, and 30 women.

### Data collection

Data were collected through FGDs between August 2023 and December 2023. FGDs effectively gather insights on health-related topics [32], foster open dialogue, and encourage in-depth discussions [33]. The FGDs were conducted by IJ (female, PhD student) and DVB (female, general practitioner and PhD student with experience in qualitative research) using a semi-structured interview guide developed by IJ. DVB conducted the first two FGDs (FGD1 and FGD2) with IJ serving as co-facilitator. Following these, IJ led the subsequent FGDs. Between the FGD sessions, DVB provided expert advice and feedback, optimizing IJ’s interview techniques. The interview guide was revised after FGD3 as part of an iterative process, based upon analyses from previous interviews. The final interview guide can be found in [additional file 2]. The FGDs were conducted on-site at the participating community organizations, ensuring a familiar and accessible setting for the participants. The point of data saturation was determined through review and discussion of topics, with data saturation reached after FGD4. The FGDs had an average duration of 1,5 h and were conducted in Dutch and French. For the Ukrainian and Arabic-speaking participants, two female volunteers, both members of the local community, joined the FGD as interpreters. One woman, a native Ukrainian speaker, served exclusively as a translator, while the other, a native Arabic speaker, acted both as a translator and as a participant. Both of them were also members of the local community. The Arabic interpreter’s dual role was chosen because, as a native speaker and community member, she provided linguistic support and culturally relevant insights aligned with the target group, enhancing the discussion’s depth. All FGDs were video- and audio recorded, transcribed verbatim afterwards and deleted upon study completion.

### Coding and data analysis

First, we analysed the data from the FGDs using the Qualitative Content Analysis (QCA) method to identify key categories. The QCA method, widely used in healthcare research [33–36], allows to get a nuanced understanding of the qualitative data. Due to the lack of a standardized methodology, procedures for QCA can differ among researchers [32, 37]. For this study, we adopted the four-step process (formulation of the research questions, sampling, coding, and analysing) proposed by White and Marsh [32]. For coding the data, we followed four steps: reading the data, open coding, axial coding, and selective coding) [31]. For the technical and procedural aspects of analysing the data in NVivo we used the methods described by Allsop et al. [38]. We started by reading the transcripts to familiarize ourselves with the data. During the step of open coding, we assigned codes that seemed relevant to answering the research question. Therefore, we used in vivo methods based on the participants’ words to stay as close to the data as possible [39]. Relevant reflections and ideas were documented in annotations while coding. Next, we performed axial coding, where we grouped these open codes into subcategories, which were defined to specify the boundaries within each subcategory. Finally, selective coding linked the subcategories to the main categories, creating a codebook. Initial coding was conducted by IJ. To enhance trustworthiness, two transcripts were randomly selected. IJ and DVB reviewed these transcripts by listening to the audio recordings and independently re-coding the FGDs. Codes and themes were subsequently discussed, with any disagreements resolved through deliberation. IJ then proceeded to code the remaining transcripts, consulting DVB periodically to review and refine the coding process, which resulted in the development of the final codebook. The final codebook is available in [Additional File 3].

**Fig. 1:**
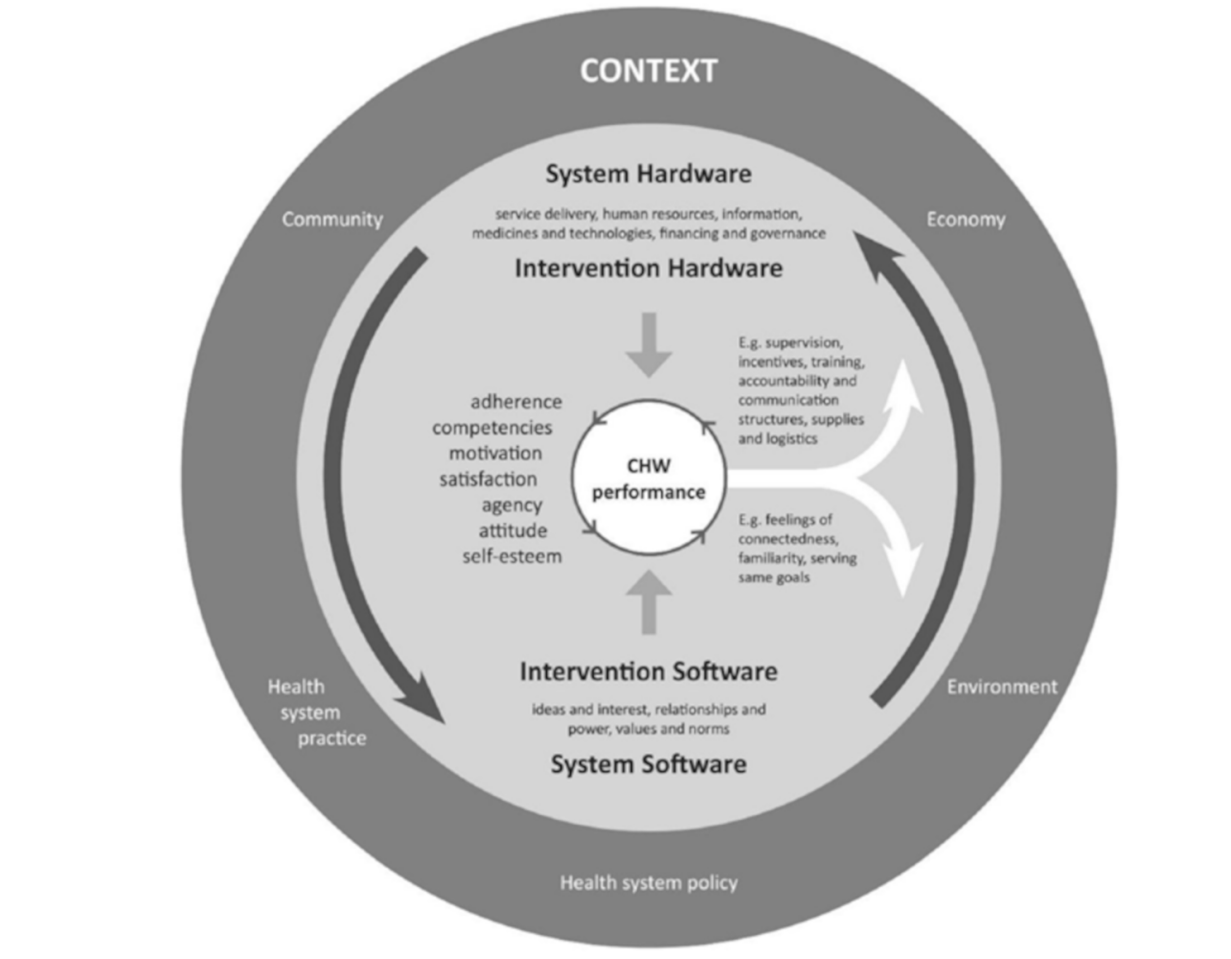
Framework of CHW performance. Adapted from Kok et al. [39], under the Creative Commons Attribution 4.0 International License (http://creativecommons.org/licenses/by/4.0/). No changes were made

Secondly, to gain deeper insights from our QCA, the findings were subsequently coded according to the attributes defined in the framework of CHW performance by Kok et al. [40] (see Fig. 1). This framework was specifically designed to examine the factors influencing CHW performance. It is worth noting that Kok’s framework adopts a CHW-centric perspective rather than an end-user perspective. Despite this distinction, the framework’s structured approach and comprehensive elements proved effective in systematically organizing and presenting the findings of this study.

Central to the framework are seven key attributes that define CHW performance at the individual level: adherence, competencies, motivation, satisfaction, agency, attitude, and self-esteem. These attributes are essential in shaping how CHWs operate and interact within these levels, where ***adherence*** is defined as the extent to which CHWs comply with established guidelines, protocols, and procedures. In the context of breast cancer screening education, adherence ensures the quality and safety of education and support. ***Competencies*** are defined as the knowledge, skills, and attitudes necessary for CHWs to fulfil their roles effectively. These are developed through training and experience. ***Motivation*** is defined as the driving force behind CHWs’ commitment to their tasks, which may stem from personal satisfaction, recognition, financial incentives, or a sense of responsibility. ***Satisfaction*** is defined as CHWs’ contentment with their working conditions, support, and recognition, which directly impacts their retention and motivation. ***Agency*** is defined as the CHW’s ability to act independently within their role and their capacity to empower communities to take collective action for health improvements. ***Attitude*** is defined as CHWs’ beliefs, values, and perspectives on their work, the target population, and the program. A positive attitude enhances motivation and effectiveness. ***Self-esteem*** is defined as the CHWs’ confidence in their abilities and the value of their work. Self-esteem is essential for managing challenges and performing effectively. We analysed the data in this study to explore participants’ views on these key attributes to identify the attributes that align most closely with the preferences of the target population regarding the ‘ideal’ profile of a CHW involved in breast cancer screening education.

### Ethics

#### Ethics approval

was received from the Medical Ethics Committee affiliated with Ghent University and Ghent University Hospital (Ref. ONZ-2023-0032) before study commencement. Before each FGD, participants received an oral review of the consent form, followed by written informed consent, which was obtained from all participants after a thorough explanation of the study protocol. Participants were informed that confidentiality would be guaranteed, transcriptions would be pseudonymized, securely stored during the study, and recorded audio and video files would be deleted upon study completion.

## Results

### Participant characteristics

In this study, we focused on recruiting women who are part of the target group for PMSP (aged 50–69). We specifically asked the organizations to include women who are less likely to participate in the PMSP, resulting in 27 women who participated across the four FGDs. Despite this request, a few participants did not fully meet the intended target group criteria. These participants were primarily volunteers from within those community organizations, whose valuable insights stem from their continuous involvement with the target population. Their inclusion was maintained because their close association with the target group provides meaningful contributions. Conversely, participants younger than 50 years (*n* = 2), were excluded from the analysis, as they lacked sufficient exposure to the lived experiences of the target group. This approach allowed us to focus on individuals who are closely aligned with the target group while recognizing the contributions of those who, although partially meeting the criteria, provide valuable contextual insights.

Furthermore, one participant (Participant 15), a volunteer from one of the participating organizations, took part in both FGD2 and FGD3, contributing to a total of 24 unique participants for analysis as presented in Table 1. The participants spoke Dutch, French, Ukrainian, or Arabic.

**Table 1:** Overview of women participating in the FGDs

Participant	Focus group discussion	Role/group type
Participant 1	FGD1	Woman from the target group
Participant 2	FGD1	Woman from the target group
Participant 3	FGD1	Woman from the target group
Participant 4	FGD1	Woman from the target group
Participant 5	FGD1	Woman from the target group
Participant 6	FGD1	Woman with a close connection to the target group
Participant 7	FGD2	Woman from the target group
Participant 8	FGD2	Woman from the target group
Participant 9	FGD2	Woman from the target group
Participant 10	FGD2	Woman from the target group
Participant 11	FGD2	Woman from the target group
Participant 12	FGD2	Woman from the target group
Participant 13	FGD2	Woman from the target group
Participant 14	FGD2	Woman from the target group
Participant 15	FGD2/FGD3	Woman with a close connection to the target group
Participant 16	FGD3	Woman with a close connection to the target group
Participant 17	FGD3	Woman with a close connection to the target group
Participant 18	FGD3	Woman from the target group
Participant 19	FGD3	Woman with a close connection to the target group
Participant 20	FGD4	Woman from the target group
Participant 21	FGD4	Woman from the target group
Participant 22	FGD4	Woman from the target group
Participant 23	FGD4	Woman from the target group
Participant 24	FGD4	Woman from the target group

### Interview findings

A range of categories emerged from the FGDs, as outlined in the codebook provided in [additional file 3]. These categories have been subsequently organized based on the key attributes outlined in the framework of CHW performance by Kok et al. [40].

## Competencies

As outlined in the framework, competencies encompass the knowledge, skills, and attitudes necessary for CHWs to effectively fulfil their roles. During the FGDs, participants emphasized that the desired competencies of CHWs are multifaceted, including professional knowledge and expertise, personal experience in shaping competencies, effective communication skills, cultural awareness and motivational and supportive competencies.

### Professional knowledge and expertise

Formal qualifications, practical experience, and a thorough understanding of breast cancer screening were identified as essential elements of the competencies required for a CHW to build trust and provide high-quality support. To support this, participants emphasized that CHWs should possess a solid (para)medical background. Although a formal medical degree was not considered essential, participants underscored the importance of CHWs’ knowledge of breast cancer care. Suggested qualifications included general practitioners, retired physicians, gynaecologists, psychologists, and specialized nurses, with experience in oncology being particularly valued. As one participant remarked:

*“Yeah*,* if it’s truly a professional who knows what they’re talking about” (Participant 13).*

Furthermore, participants emphasized that a CHW with professional training in the field would inspire greater confidence compared to someone lacking such expertise:

*“Yeah*,* I can’t really*,* he might not have enough knowledge about it. Except if it’s truly someone professional*,* who is continuously involved in it. That inspires more*,* um*,* confidence than someone who just briefly […]” (Participant 13).*

At the same time, participants also acknowledged that practical experience and understanding could also suffice if the CHW had the right knowledge and skills. Whether acquired through formal education or alternative training, having the necessary expertise was deemed essential. One participant remarked:

*“A kind of, an education and yes, the right knowledge, having followed the education or similar, but she must have the knowledge” (Participant 21– translation for participant 22).*

### Personal experience in shaping competencies

In addition to professional knowledge, personal experience with breast cancer was highlighted as an important role in shaping the competencies of CHWs. Participants emphasized the value of CHWs who have undergone mammograms or who have direct experience with breast cancer, as this fosters both empathy and a deeper understanding of the participants’ journey. Personal experience, often associated with age, was considered a key competency, with participants highlighting that a CHW who had experienced breast cancer firsthand could offer more relatable guidance. One participant noted:

*“Yes. If she has experienced it, then it’s good. Then yes. Then she can at least say okay, what she experienced. Does it hurt or not, it’s more about the feelings themselves. So, she can explain it better. But for example, someone who hasn’t experienced it, for her, no. No way” (Participant 21 - translation for Participant 24).*

Another participant elaborated on the value of such experience, describing how a CHW who had been through a mammogram and subsequent diagnosis could offer valuable insights:

*“It should be someone who has experienced it*,* who has had a mammogram done. But through*,* thanks to the mammogram*,* they found something*,* a lump. And thanks to that lump*,* she was treated in time*,* promptly*,* actually*,* and that’s important and*,* and […]. That’s actually something that should be discussed” (Participant 21 - translation for Participant 24).*

Additionally, participants noted that CHWs with relevant personal experiences were uniquely positioned to provide empathetic, practical support. One participant described how a CHW’s ability to draw on lived experience enhanced the depth of guidance they could offer:

*“I think the most important thing is*,* has that person experienced something? Because if you have that examination*,* and you receive the bad news*,* that person can maybe still help you in some way*,* to tell you about the support and what comes next*,* what happens. And not just reading a text like*,* now this will happen and then that will happen.” (Participant 20).*

### Effective communication skills

Effective communication emerged as another crucial component of CHW competencies. Participants highlighted that strong communication skills are vital for CHWs to build trust and to convey information clearly. Specifically, they emphasized that CHWs should be approachable and capable of initiating conversations in a non-intimidating manner. This aligns with the need for CHWs to possess the skills to explain complex topics, such as the risks and benefits of breast cancer screening, while fostering a sense of ease and understanding. As one participant explained:

*"Just, you know. Just talking, social, just, right? Not, um, showing, I know more than you, because I’m here to explain everything to you" (Participant 4).*

Equally important is the CHW’s capacity to adapt their communication style to meet the needs of diverse audiences. This includes simplifying complex health information to ensure clarity and accessibility. Participants highlighted the importance of situational awareness and flexibility in communication, as illustrated by participant 4:

*"Just, like, when you arrive at someone’s place, you start explaining a few things and that person starts looking at you like this, so then you have to say ‘click’ in your head like ‘oh, what I’m explaining right now, that person doesn’t understand’. So, then you have to lower your level yourself and try to speak with ordinary words or with a little drawing or whatever, so that person really understands what you’re actually here for" (Participant 4).*

### Cultural awareness

Cultural awareness was also deemed an essential component of competencies, emphasized by participants as reflecting the CHW’s ability to engage effectively with diverse communities, with a particular focus on the importance of being attentive to cultural differences, as demonstrated in the following quote:

“It should be someone who is also aware of potential cultural differences and takes them into account” (Participant 6*).

### Motivational and supportive competencies

Participants highlighted essential competencies that CHWs need to successfully fulfil their dual role as providers of emotional support and guidance. These include the ability to motivate and encourage individuals to participate in breast cancer screening programs, positively influencing their decision-making. One participant stressed the significance of this skill, noting that CHWs could “motivate” and “support” individuals in undergoing mammography screening. This emphasizes CHWs’ role in promoting proactive health behaviours. As another participant stated:

*“Because actually*,* that also motivates us to go*,* and it’s actually like an additional push*,* a kind of push that she’s actually going to give us to also uhm*,* have a further examination*,* well*,* a mammography*,* a push to do the screenings ourselves” (Participant 21).*

## Attitude

The attribute attitude closely aligns with the perspectives of the target population regarding the CHW profile. The FGDs illustrate how various aspects of attitude, such as patience, empathy, sociability, flexibility, and impartiality, are crucial in fostering effective and supportive relationships with the target group.

### Patience

Participants highlighted the importance of patience as a key attitude for CHWs, often associating this attitude with age. Some participants expressed a preference for older CHWs, approximately 50 years of age, attributing their patience to greater life experience. Others, however, favoured younger CHWs, aged between 20 and 30, suggesting that they too could demonstrate patience effectively. As one participant explained:

*“She prefers the 50-year-olds because someone has actually experienced something in life already*,* whereas she says “yes*,* we don’t have patience anymore*,* between 20 and 30*,* those are the persons who haven’t actually experienced much yet*,* so who still actually have patience” (Participant 21 - Translation of a conversation between participant 22 and 24).*

### Manners and empathy

Participants stressed the importance of good manners, particularly politeness, in all CHWs interactions. A CHW was expected to be kind, friendly, warm, calm, understanding, gentle, resilient, and, above all, empathetic. As one participant stated:

*“I also think of someone who understands your emotions*,* your anxiety*,* that*,* that is important” (Participant 20).*

### Sociability and flexibility

Several participants highlighted the need for a CHW to be *“extremely sociable”* and adaptable, capable to engage comfortably with people from different backgrounds. This adaptability reflects a positive attitude towards diversity and the ability to tailor communication to the unique needs of individuals. As one participant stated:

“Flexible? Yes, because they will have to deal with a lot of different people. (…) Both with more difficult people and with, well, yes. That they can adapt to anyone” (Participant 15*).

Another participant emphasized the importance of adaptability in varying social contexts:

*“Take, for example, the comparison between a row house and a large villa. Well, it’s a very good comparison. They belong to two different categories. People aren’t bad, but they simply have a different attitude, do you understand? Therefore, the CHW must be able to adapt” (Participant 18).*

### Impartiality and nonjudgmental listening

Another important aspect discussed was the need for the CHW to maintain impartiality, free from prejudice. As one participant noted: *“Someone who also listens*,* but who also won’t judge*,* like ‘this or that’*,* but who really just listens” (Participant 20).* In addition, participants highlighted the importance of the CHW’s attentiveness as a listener, with *participant 20* stating that the CHW should be *“A listening person”.*

## Agency

The data from the FGDs emphasize the importance of CHWs demonstrating a strong sense of autonomy and assertiveness. Participants highlighted that CHWs should not easily be influenced or undermined by others, stressing the importance of maintaining professional boundaries. As one participant noted: *“The CHW should also not be someone who just allows themselves to be taken advantage of” (Participant 18)*, while another stated, *“Let them not be walked all over” (Participant 19).* These quotes underscore the value placed on CHWs who exhibit confidence and strength, ensuring they are not dismissed or disregarded, particularly when conveying breast cancer screening-related information.

Furthermore, participants emphasized the importance of CHWs being connected to a professional network, such as a hospital, as this would allow them to provide accurate and reliable information. This professional network was viewed as essential for facilitating CHWs’ ability to guide individuals within the healthcare system effectively. These findings align closely with the conceptualization of agency in Kok et al.‘s model, wherein CHWs demonstrate the ability to act autonomously while maintaining connections with professional support structures, thereby enhancing their capacity to empower communities and ensure the delivery of quality health care. One participant articulated this need as follows:

*“(…) the people from the clinic*,* so working together with the people from the clinic well. And a bit*,* because she says*,* yes*,* they are nice people*,* but yeah*,* we also don’t know that for example” (Participant 21).*

## Adherence

Participants emphasized the importance of CHWs adhering to established guidelines and protocols when delivering information. Adherence ensures that CHWs provide accurate, consistent, and reliable information, which is critical for the credibility of their role. Moreover, adherence to guidelines was associated with participants’ expectations for clear communication and the CHW’s ability to respond to questions in alignment with established procedures. Participants expressed that the CHW should be capable of offering further clarification when additional questions arise regarding the screening process. One participant specifically noted:

*“If there are people who have questions about it*, that it’s someone who can also provide further explanation about it” (Participant 6*).

## Self-esteem

Participants emphasized that CHWs should demonstrate confidence in their knowledge and communication skills, as this was seen as essential for effectively delivering breast cancer screening education. One participant highlighted the importance of confidence by stating:

*“Yeah*,* she just needs to know where she’s coming from*,* what she’s talking about.” (Participant 21 - Translation of a conversation between Participant 22).*

This sentiment was broadly echoed across multiple focus group discussions, where participants collectively expressed:

"The CHW should be someone who just says what he or she needs to say (Participant 5), and who knows what he or she is talking about” (Participant 15*, 13,19, 22, 24).

These results underscore the perceived importance of self-esteem, as reflected in the target group’s emphasis on CHWs demonstrating confidence in their knowledge and abilities. Such confidence is viewed as essential for effectively delivering breast cancer screening education and addressing challenges inherent to the CHW’s role. This aligns with Kok et al.‘s framework, which highlights the key function of self-esteem in shaping CHWs’ effectiveness in empowering communities and delivering high-quality care.

## Trust as the cornerstone

Throughout the FGDs, trust emerged as a central theme, serving as a cornerstone in bridging CHWs attributes with their perceived effectiveness in engaging with the target group. Participants highlighted that trust is shaped by a combination of key enabling factors, including age, familiarity and personal connections, relational skills, and gender. These factors play a crucial role in influencing how trust is developed and maintained within community health interactions.

### Age

Trust was frequently linked to the CHW’s age, were some participants were more likely to trust older CHWs, with some participants acknowledging that they would doubt the information provided by younger CHWs. A preference for CHWs aged between 20 and 60 years was commonly expressed, with age perceived as a factor influencing both personal experience and trustworthiness. Participants also linked trust to the CHW’s personal experience with mammography, with one participant emphasizing the role of personal experience in building trust:

*“No*,* I actually don’t think age is important. I think the most important thing is*,* that*,* that person has been through something?” (Participant 20).*

Another participant emphasized the value of having a CHW perceived as knowledgeable and experienced, noting that such an individual would be viewed as more trustworthy in conveying accurate information.

*“Firstly*,* because I know that person has had it herself and that person knows about what she is doing and how that can make you feel*,* and I would have enormous confidence in such a person who then wants to explain to me about breast cancer” (Participant 4).*

### Familiarity and personal connections

Familiarity with the CHW was also emphasized as a key factor in building trust. Participants noted that personal relationships and previous interactions with a CHW play a significant role in establishing trust. For example, one participant stated:

*“As I mentioned*,* I think of someone we know*,* with whom we have already had contact. We are all here together*,* perhaps someone we have already met” (Participant 14).*

This highlights how familiarity enhances trust, a crucial aspect of the CHW’s competencies in fostering meaningful interactions. Moreover, participants stressed the importance of personal connections, such as knowing someone with experience of breast cancer, as a factor that strengthens their trust in the CHW. One participant commented:

*“But in my mind*,* there’s still*,* well*,* something lingering that makes me think*,* ‘I hope that person knows someone in their environment or family who has had breast cancer.’ Because then I’ll trust that person more*,* that’s how I feel*,* that’s my opinion*,* I have more trust in that person*,* then in someone who has graduated and yes*,* who receives training there and that and that” (Participant 4).*

### Relational skills

Relational skills were also identified as a crucial aspect of the competencies required for CHWs to effectively fulfil their roles. These skills encompass the ability to foster supportive and trusting relationships, which are essential for engaging with individuals in a way that builds trust and provides reassurance. Participants highlighted that CHWs must possess the practical ability to make individuals feel comfortable and at ease, emphasizing that such relational skills are a core component of their role. As one participant noted:

*“Having someone who guides you and also reassures you” (Participant 20). Another stated*,* “So*,* to put us at ease*,* to make us calm. They must have experienced that” (Participant 21).*

### Gender

During the FGDs, most participants emphasized the importance of the CHW’s attitude in fostering trust and a sense of safety, particularly in the context of breast cancer screening. Gender was highlighted as a significant factor influencing these attitudes. Participants frequently used terms such as “woman,” “madam,” “femme,” or “helper” (explicitly referring to a female helper), as well as feminine pronouns like “she” or “her” when discussing the ideal CHW. Many believed that a female CHW’s attitude naturally aligns with the sensitivity and empathy required for addressing sensitive health matters, such as breast cancer screening. One participant remarked:

*“Gosh, when it comes to breast cancer, I prefer a woman anyway” (Participant 4).*

Another shared:

*“Yes. Although I prefer to go to a male gynaecologist*,* but personally if it is for me*,* well*,* in such a project*,* I would rather prefer it to be a lady” (Participant 18).*

However, some participants did not express a gender preference or indicated a preference for a male CHW. One participant highlighted that men are perceived as “serious people” and “born researchers,” suggesting a belief that professionalism and expertise might be prioritized over gender. Another participant noted that the gender of the CHW would not be a determining factor:

*“Giving the information. For me, that’s actually equal then a man or a woman” (Participant 10).*

## Discussion

The integration of CHWs has proven to be an effective strategy for addressing disparities in access to care and participation in breast cancer screening programs [15–26]. However, many studies fail to explicitly define the (preferred) profile of CHWs, making it challenging to evaluate how these characteristics influence their performance. Research has indicated that CHW performance can be shaped by characteristics such as gender, age, and personal experiences [41]. However, these characteristics are frequently overlooked during the design and implementation of an intervention. Notably, the ‘ideal’ profile for CHWs involved in breast cancer screening education remains undefined. This study seeks to address this gap by exploring the preferences of women of the target group regarding the desired profile of CHWs involved in breast cancer screening education. Using QCA, key categories were identified and subsequently organized within the framework of CHW performance developed by Kok et al. [39]. This approach provided nuanced insights into participants’ preferences for CHWs in the context of breast cancer screening education and allowed us to identify the preferred attributes that resonate most with the target population.

The findings emphasize the importance of CHW competencies, including professional expertise, personal experience, and effective communication skills. Within the competency attribute, several conflicts emerged regarding participants’ preferences, highlighting the nuanced expectations regarding the preferred competencies a CHW should possess. Participants not only expressed a strong preference for CHWs with professional knowledge, but also for CHWs with personal experiences, such as having undergone mammography or having a personal history with breast cancer. This dual competency, combining both professional knowledge and personal experience, was perceived as crucial for fostering trust, empathy, and effective communication. Additionally, participants underscored the importance of CHWs possessing specific knowledge about breast cancer and the screening process, complemented by the ability to communicate this information clearly, comprehensibly, and with compassion, ensuring that individuals feel understood and supported throughout their breast cancer screening journey. These results challenge the existing literature, which tends to prioritize formal training and technical knowledge [17] rather than knowledge gathered through personal experiences. Participants in this study valued knowledge gathered through personal experiences equally, if not more, compared to formal training and technical knowledge. The participants’ preference for CHWs with personal experiences reflects a perception that such experiences enhance credibility and offer emotional support, elements often lacking in traditional healthcare approaches. To our knowledge, existing research has primarily focused on CHWs’ formal training and technical expertise related to breast cancer, screening guidelines, and mammography [15, 17, 19, 24, 42], while overlooking how personal experiences might complement professional knowledge. This study suggests that combining personal experiences with formal training could offer comprehensive and compassionate support, particularly for women who are hesitant to participate in, or do not participate in screening programs. However, recruiting CHWs with both professional expertise and personal lived experience, such as having undergone mammography or having a breast cancer history, presents multiple challenges. The pool of candidates with formal training is often limited and may skew toward highly educated individuals who lack the close community ties or lived experience valued by our participants. Conversely, those with relevant lived experience from within the community might not possess the professional qualifications required, necessitating tailored training programs that increase costs and extend preparation time. Finding and preparing these CHWs can take considerable time, which might delay program implementation. Moreover, once recruited, balancing these dual roles could strain CHWs both professionally and emotionally. CHWs must deliver accurate health information and adhere to guidelines, while their lived experience might lead them to share personal insights, whether from screening or a cancer journey, to foster trust and empathy, though this is not mandatory. Such sharing could prompt them to revisit personal challenges, creating tensions with professional objectivity and risking emotional exhaustion. Over time, this strain might diminish CHWs’ effectiveness or lead to withdrawal, threatening program sustainability through high turnover or disrupted community support. Addressing this requires not only careful selection but also robust support systems, such as counselling or peer networks, to help CHWs manage these pressures and maintain their well-being.

Beyond competencies, participants emphasized the importance of CHWs’ attitudes, such as empathy, sociability, and adaptability to diverse personalities and backgrounds. These attitudes were seen as essential for fostering a safe and supportive environment, as they enable CHWs to build trust and effectively interact with individuals from diverse backgrounds. Attitudes, therefore, can be seen as a critical enabler of competencies, enhancing the overall impact of CHWs’ work in addressing community needs. Furthermore, participants identified trust as a central theme. Participants stressed that CHWs should be familiar with the community, culturally sensitive, and capable of establishing personal connections. A deep understanding of the community’s social and cultural contexts, along with prior positive interactions, was considered vital for effective engagement. In addition, gender emerged as a key factor influencing participants’ perceptions of the ideal CHW’s, particularly regarding attitudes such as sensitivity and empathy. These attitudes were seen as essential for fostering trust and a sense of safety, especially in the context of addressing sensitive health topics like breast cancer screening. The majority expressed a preference for female CHWs, frequently associating femininity with empathy, sensitivity, and the ability to address delicate health issues. This aligns with findings in the literature, where female CHWs are often employed within breast cancer screening education to enhance comfort and trust [16, 17, 41]. In addition, this preference for female CHWs, tied to empathy and trust, aligns with patterns seen in European contexts where community ties shape CHW roles in breast cancer initiatives. For instance, a review by Van Iseghem et al. [12] shows how Belgian screening programs rely on CHWs’ cultural sensitivity and community familiarity to foster trust and encourage participation, much like the attitudes valued in this study. Similarly, in a study from eastern Turkey [19], female peer educators build trust by addressing cultural health beliefs in a conservative setting, echoing the empathy and gender-based comfort participants preferred in this study. Still, differences stand out: in this study participants prized psychological rapport and personal trust rooted in individual connections, whereas Belgian screening programs, as described by Van Iseghem et al. [12], focus on fostering trust through wider community engagement, and the eastern Turkish approach, as described by Gözüm et al. [19] leans on aligning with conservative norms rather than emphasizing personal experiences. These contrasts highlight how trust and gender remain central, yet the ideal CHW profile adapts to local expectations, favouring personal rapport in this study’s Western European context, while reflecting cultural traditions elsewhere when needed. Nonetheless, a minority of participants prioritized expertise over gender, with some expressing a preference for male CHWs, perceived as serious and professional.

In addition, participants highlighted that CHWs should fulfil roles that go beyond providing information. The participants expressed a desire for CHWs to provide emotional support and guidance, emphasizing the importance of their role in facilitating informed decision-making and addressing emotional concerns as part of their competency traits. Emotional intelligence was deemed essential for creating a supportive environment where women feel heard and reassured. Many participants viewed CHWs as key motivators who could offer the encouragement needed to overcome hesitations about breast cancer screening.

The findings in this study align with, and further elaborate on the CHW performance framework. Within this framework, the attributes of CHW performance are derived from existing literature, reflecting CHWs’ perspectives, focusing on their roles, experiences, and needs, rather than being directly assessed from the perspectives of the end users. The framework is based on a systematic review of 140 CHW programs, a qualitative study across four African countries, existing health worker performance models, the concept of health systems as social constructs, and the WHO health systems framework [39]. While comprehensive, this approach does not address the impact of CHW performance on end users and the communities they serve. By integrating the QCA-derived categories into the framework, this study bridges that gap, providing actionable insights to align CHWs’ attributes with women’s preferences. While this perspective provides valuable insights, it is important to recognize that our findings primarily reflect the perceptions of the target group rather than the lived experiences of CHWs themselves, limiting our scope to explore certain attributes from the framework of Kok et al. like motivation and satisfaction directly. Still, these attributes appear relevant to the preferences we identified. For instance, motivation, defined as the driving force behind CHWs’ commitment, shaped by factors like recognition or responsibility—could strengthen their ability to build trust and show empathy, qualities our participants valued highly. Similarly, satisfaction, linked to contentment with support and working conditions, might enhance CHWs’ capacity to adapt and communicate effectively, traits our target group preferred. These connections align with Kok et al.’s emphasis on motivation and satisfaction, as drivers of retention and performance, though they remain speculative here due to the absence of CHW-specific data. Further research is needed to test these potential links and their impact on end-user needs, as our study does not empirically assess what motivates or satisfies CHWs. Instead, the results shed light on how the target group perceives and values certain CHW attributes, such as empathy, cultural understanding, and personal involvement with the topic. These qualities were emphasized by participants as key to building trust and credibility in CHW interactions. This distinction underscores a limitation in the alignment between our research question and the framework’s CHW-centred approach. While the framework focuses on CHWs’ intrinsic attributes, our study highlights the expectations and interpretations of the end users. These perspectives are complementary rather than contradictory. By integrating such end-user insights, the framework could offer a broader understanding of the interplay between CHWs’ intrinsic motivations and target group-focused expectations. Importantly, it emphasizes that attributes such as motivation and satisfaction, when viewed through the lens of end users, are not direct observations but rather interpretations of what communities’ value in their interactions with CHWs. This complementary perspective does not seek to replace CHWs’ lived experiences but rather enriches the framework, offering practical guidance for tailoring CHW recruitment, training, and support to better align with both CHWs’ professional needs and the expectations of the communities they serve.

Furthermore, a notable methodological strength of this study is its focus on end-user preferences in a context like Ronse, where CHWs are not yet established, allowing participants to conceptualize an ideal CHW profile without preconceived biases. However, the lack of familiarity with CHWs made it challenging for participants to envision a role or profile for a CHW that does not yet exist, potentially limiting their ability to fully articulate the contributions CHWs could make to breast cancer screening education. Moreover, discussing this topic may have evoked emotionally charged responses, such as feelings of judgment or guilt toward women hesitant to participate in screening, which could have influenced how participants described CHWs and their profiles. These methodological considerations are further compounded by the study’s reliance on a small, localized sample from the city of Ronse. While this focus provides valuable insights into the preferences of women in this specific context, it may limit the generalizability of the findings to other regions or populations in Flanders with differing sociodemographic profiles or to regions where CHW programs are already established. Further research is needed to explore how these findings translate to different settings and demographic contexts, including other regions in Flanders to enhance broader applicability.

Furthermore, while the use of FGDs offered valuable qualitative insights into participants’ perspectives, it is important to acknowledge potential limitations inherent in the methodology. The group setting may have introduced social desirability bias, potentially influenced participants’ responses and shaping the data collected. Group dynamics in FGDs might have influenced responses, such as their expressed views, preferences or stance toward breast cancer screening, due to peer pressure, dominant voices, the silencing of quieter voices or conformity, all known limitations of this method [43]. To mitigate these effects, we implemented strategies such as ensuring a limited hierarchy among participants (e.g., by recruiting peers with similar backgrounds) and having moderators actively encourage quieter voices while tempering dominant ones. Furthermore, the inclusion of organizational volunteers, predominantly in one focus group (FGD3), enriched contextual insights due to their strong ties to the target group but may introduce selection bias. These volunteers, often screened for breast cancer unlike typical non-participants, may not fully reflect the broader PMSP non-participant group due to their engagement with community services. In our analysis, we used analytical memos to separate their personal views from those representing the target population, cross-checking their insights in later focus groups to confirm relevance to the wider group. Moreover, the use of volunteer interpreters, including one who also participated, may risk misinterpretation. Professional interpreters could improve accuracy in future studies. Additionally, the inductive analytical approach used in this study was effective for identifying emergent categories, which were subsequently mapped onto the framework. However, this method limited the extent to which certain attributes from the framework were discussed or left unaddressed. To address this limitation, future research could adopt a more deductive approach from the outset. This would ensure that all attributes of the framework are systematically explored, providing a more comprehensive understanding of end-user preferences and further enhancing the framework’s applicability in guiding the selection and training of CHWs in breast cancer screening education.

## Conclusions

This study explored the preferences of women in the city of Ronse, who are less likely to participate in the PMSP, regarding the ideal profile of CHWs for breast cancer screening education. Key findings reveal that a combination of professional knowledge and personal experiences, such as having undergone mammography or breast cancer treatment, is critical for building trust and fostering effective communication. Participants emphasized the importance of CHWs demonstrating empathy, sociability, and adaptability to diverse personalities and cultural contexts. In addition, gender emerged as a key factor influencing participants’ perceptions of the ideal CHWs, with female CHWs being preferred due to their perceived empathy, sensitivity, and ability to foster trust and a sense of safety in addressing sensitive health topics such as breast cancer screening.

The findings of this study provide actionable insights for recruiting and training CHWs, underscoring the need for a balanced combination of technical knowledge, personal experience, and interpersonal skills to address the specific preferences of the target population. By integrating end-user perspectives, this research bridges gaps in existing CHW performance frameworks, offering a more comprehensive understanding of CHW performance. Rather than attempting to define CHWs’ attributes and how they influence their performance, it focuses on how these attributes are perceived and valued by the target group. This approach enhances the relevance of existing frameworks by complementing them with insights into the expectations and experiences of the communities served. These findings support the effective design and implementation of CHW programs tailored to the unique requirements of target population. While these findings offer valuable guidance, recruiting and training CHWs with the ideal combination of competencies, personal attributes, and life experiences remains a notable challenge. Addressing this challenge will require innovative approaches to CHW selection, training, and support to ensure the success of breast cancer education programs. Training CHWs with this ideal profile could involve several actionable steps. First, partnerships with breast cancer survivor networks could facilitate the recruitment and training of individuals with relevant lived experience. Second, collaborating with healthcare institutions could provide access to short, targeted courses on breast cancer screening protocols, ensuring CHWs gain essential technical knowledge. Third, incorporating role-playing exercises led by communication experts could strengthen CHWs’ ability to convey information clearly and compassionately, addressing diverse community needs. These steps aim to bridge the gap between formal training and personal experience, equipping CHWs to meet the target group’s expectations effectively. Further research should explore these findings in other contexts to validate their broader applicability and refine CHW selection criteria to maximize their impact on target groups.

## Electronic supplementary material

Below is the link to the electronic supplementary material.

Supplementary Material 1

Supplementary Material 2

Supplementary Material 3

## Data Availability

The datasets used and/or analysed during the current study are not publicly available. All data are centrally stored on the server of Ghent University (Belgium). All data was anonymized at Ghent University, and all raw data that could lead to the identification of the participants was permanently removed. The non-identifiable datasets used and/or analysed during the current study are available from the corresponding author on reasonable request.

## Abbreviations

PMSP: Population-based Mammography Screening Program
WHO: World Health Organization
CCD: Centre for Cancer Detection
CHW: Community Health Worker
QCA: Qualitative Content Analysis
FGD: Focus Groups Discussion
