# Supplementary material for: Understanding the profile of community health workers in breast cancer screening education: women’s preferences and insights from a qualitative focus group study
Source: Int J Equity Health. 2025 Jul 4;24:193. doi: 10.1186/s12939-025-02508-0 (PMC12231706; doi:10.1186/s12939-025-02508-0)
Supplement: Supplementary file 1 — Supplementary Material 1 [file 12939_2025_2508_MOESM1_ESM.docx]

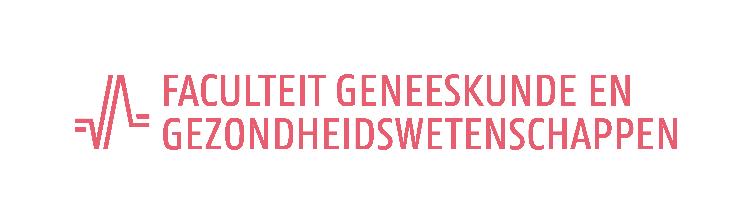

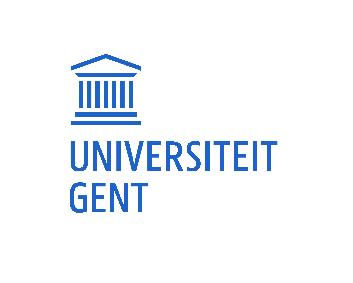


# Interviewguide – Part 1

**"Understanding the Profile of Community Health Workers in Breast Cancer Screening Education: Women's Preferences and Insights from a Qualitative Focus Group Study"**

**Introduction:**

Let me first introduce ourselves. I am Ilka Jacobs, and this is Dorien Vanden Bossche, and we are researchers at the University of Ghent.

I have gathered you here today because Flanders is one of the regions with the highest number of new breast cancer cases in Europe. As a result, women between the ages of 50 and 69 receive an invitation every two years from the breast cancer screening program (the government) to undergo a free mammogram. Nearly half of the women (44.7%) in Ronse who have received this invitation have not responded to it and have therefore not had a mammogram.

I (Ilka) would like to investigate what factors prevent women from responding to this invitation and how I can improve this situation.

- **"Is this clear to everyone, or are there any further questions or concerns about this?"** *(Wait before proceeding)*

Previous research has shown that women are often not sufficiently informed about participating in the breast cancer screening program, or they do not understand the invitation letter and/or think it is just advertising.

Therefore, I (Ilka) would like to use 'Community Health Workers (CHWs)' in my research to spread more information about breast cancer screening. A CHW is someone from your community who has received short training to help you and others. A CHW can offer various types of assistance, such as providing information about breast cancer screening, helping you make an appointment, or ensuring that you receive the appropriate care (for example, by accompanying you to the mammogram).

These Community Health Workers (CHWs) are currently not well known in Belgium. We are still in the process of determining which qualities a CHW should have in order to provide you and others with the best possible information about the breast cancer screening program, breast cancer screening, and everything related to it.

- **"Is this clear to everyone, or are there any further questions or concerns about this?"** *(Wait before proceeding)*

And this is where I need your help today!

With today's group discussion, I would like to know what you are looking for in a CHW, what makes a good CHW in your opinion, and what qualities (these can be both external and internal traits) a CHW should have so that you feel comfortable with that person.

Additionally, I would also really like to know what information you would like to receive from a CHW to ensure that you have enough knowledge about participating in the breast cancer screening program, breast cancer screening itself, and so on.

With all the information gathered today, we will be able to select the right CHWs who can inform women about breast cancer screening.

I personally believe it is very important that women receive sufficient information about the breast cancer screening program, the invitation letter, and the screening process so that they can make an informed decision about whether or not to participate in breast cancer screening.

- **"Is this clear to everyone, or are there any further questions or concerns about this?"** *(Wait before proceeding)*

Before we begin the group discussion, I would like to provide some more information about my research and go over this information letter with you.

By signing this letter, you give your consent to me (Ilka) and my research team to use everything discussed here today for my study.

**[Go over the information letter + have it signed]**

- **"Is this clear to everyone, or are there any further questions or concerns about this?"** *(Wait before proceeding)*

# Interviewguide – Part 2

**Opening question:**

- **"Can you briefly introduce yourself?"**

**Introductory Question:**

- **"When you think of a CHW, who do you think of? How would you describe this person?"**

**Key questions**

- **"What characteristics/traits should a CHW possess?"**
- **"What skills should a CHW have?"**
- **"What makes you feel comfortable with a CHW?"**
- **"How would you like to receive information from the CHW?"** *(e.g., individually through a personal conversation, through an information session, brochures, presentations, etc.)*
- **"What information would you like to receive from the CHW about breast cancer screening?"**
- **"Should this CHW be part of a general practice or an organization like Vrolijke Kring or the University?"**
- **If it is mentioned that the CHW should be an expert by experience** 🡪 explore this further. *What is an expert by experience? Experience with breast cancer screening? Experience with breast cancer? Experience with social poverty? Other experiences? Or all three?*

**Concluding questions**

- **"There have been many characteristics/traits/skills discussed; which ones do you think are the most important?"**
- **"Is there anything we missed?"**
- **"Is there anything else you would like to add?"**

**Back-up questions**

- **"We’ve already heard about several characteristics/traits/skills. What’s your opinion on this?"** *(Give the floor to a rather quiet participant)*
- **"Thank you for your input. Would it be okay if I pass the floor to another participant for now?"** *(Addressing a particularly active participant)*
- **Profile Characteristics of CHWs in the Literature**: Language skills, educational background, life experience, work experience, migration background, age, place of residence, gender, network.
- **Ask who would like to share their contact information for a potential follow-up individual interview.**
