# Supplementary material for: Understanding the profile of community health workers in breast cancer screening education: women’s preferences and insights from a qualitative focus group study
Source: Int J Equity Health. 2025 Jul 4;24:193. doi: 10.1186/s12939-025-02508-0 (PMC12231706; doi:10.1186/s12939-025-02508-0)
Supplement: Supplementary file 2 — Supplementary Material 2 [file 12939_2025_2508_MOESM2_ESM.docx]

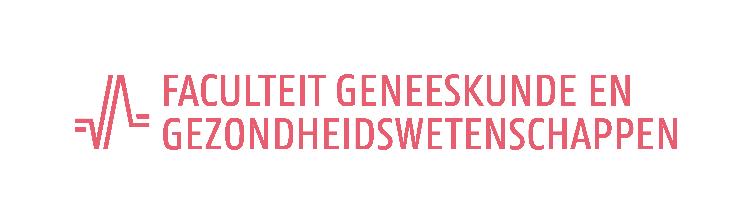

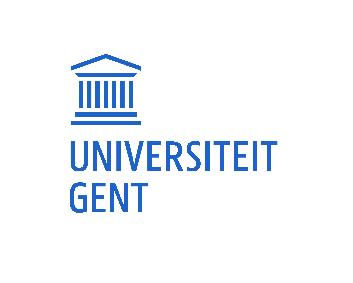


# quality assessment

**"Understanding the Profile of Community Health Workers in Breast Cancer Screening Education: Women's Preferences and Insights from a Qualitative Focus Group Study"**

## **Consolidated criteria for reporting qualitative studies (COREQ): 32-item checklist**

| **ITEM** | **DESCRIPTION** | **REPORTED ON PAGE NUMBER:** |
| --- | --- | --- |
| **Domain 1: Research team and reflexivity – Personal Characteristics** | | |
| 1. Interviewer/facilitator | Which author/s conducted the interview or focus group? | 9 |
| 2. Credentials | What were the researcher’s credentials? E.g. PhD, MD | 9 |
| 3. Occupation | What was their occupation at the time of the study? | 9 |
| 4. Gender | Was the researcher male or female? | 9 |
| 5. Experience and training | What experience or training did the researcher have? | 9 |
| 6. Relationship established | Was a relationship established prior to study commencement? | 9 |
| 7. Participant knowledge of the interviewer | What did the participants know about the researcher? E.g. personal goals, reasons for doing the research | Not reported |
| 8. Interviewer characteristics | What characteristics were reported about the interviewer/facilitator? E.g. Bias, assumptions, reasons and interests in the research topic | Not reported |
| **Domain 2: study design - Theoretical framework** | | |
| 9. Methodological orientation and Theory | What methodological orientation was stated to underpin the study? e.g. grounded theory,  discourse analysis, ethnography, phenomenology, content analysis | 10 |
| 10. Sampling | How were participants selected? e.g. purposive, convenience, consecutive, snowball | 8-9 |
| 11. Method of approach H | How were participants approached? e.g. face-to-face, telephone, mail, email | 7-8 |
| 12. Sample size | How many participants were in the study? | 14 |
| 13. Non-participation | How many people refused to participate or dropped out? Reasons? | 13 |
| 14. Setting of data collection | Where was the data collected? e.g. home, clinic, workplace | 8-9 |
| 15. Presence of non-participants | Was anyone else present besides the participants and researchers? | Not applicable/  not reported |
| 16. Description of sample | What are the important characteristics of the sample? e.g. demographic data, date | 13-15 |
| 17. Interview guide | Were questions, prompts, guides provided by the authors? Was it pilot tested? | 9 |
| 18. Repeat interviews | Were repeat interviews carried out? If yes, how many? | Not reported |
| 19. Audio/visual recording | Did the research use audio or visual recording to collect the data? | 10 |
| 20. Field notes | Were field notes made during and/or after the interview or focus group? | Not reported |
| 21. Duration | What was the duration of the interviews or focus group? | 9 |
| 22. Data saturation | Was data saturation discussed? | 9 |
| 23. Transcripts returned | Were transcripts returned to participants for comment and/or correction? | Not applicable/  not reported |
| **Domain 3: analysis and findingsz – Data analysis** | | |
| 24. Number of data coders | How many data coders coded the data? | 10-11 |
| 25. Description of the coding tree | Did authors provide a description of the coding tree? | 11 |
| 26. Derivation of themes | Were themes identified in advance or derived from the data? | 10-11 |
| 27. Software | What software, if applicable, was used to manage the data? | 10 |
| 28. Participant checking | Did participants provide feedback on the findings? | Not applicable/  not reported |
| 29. Quotations presented | Were participant quotations presented to illustrate the themes / findings? Was each quotation identified? e.g. participant number | 13-28 |
| 30. Data and findings consistent | Was there consistency between the data presented and the findings? | 13-28 |
| 31. Clarity of major themes | Were major themes clearly presented in the findings? | 13-28 |
| 32. Clarity of minor themes | Is there a description of diverse cases or discussion of minor themes? | 13-28 |

# Standards for Reporting Qualitative Research (SRQR) – Guidelines

| **ITEM** | **DESCRIPTION** | **REPORTED ON PAGE NUMBER:** |
| --- | --- | --- |
| Title | Concise description of the nature and topic of the study Identifying  the study as qualitative or indicating the approach (e.g., ethnography,  grounded theory) or data collection methods (e.g., interview, focus  group) is recommended | 1 |
| Abstract | Summary of key elements of the study using the abstract format of  the intended publication; typically includes background, purpose,  methods, results, and conclusions | 2-3 |
| Introduction | | |
| Problem formulation | Description and significance of the problem/phenomenon studied;  review of relevant theory and empirical work; problem statement | 4-6 |
| Purpose or research question | Purpose of the study and specific objectives or questions | 6-7 |
| Methods | | |
| Qualitative approach and research paradigm | Qualitative approach (e.g., ethnography, grounded theory, case study, phenomenology, narrative research) and guiding theory if appropriate; identifying the research paradigm (e.g., postpositivist, constructivist/ interpretivist) is also recommended; rationale | 7-8 |
| Researcher characteristics and reflexivity | Researchers’ characteristics that may influence the research, including  personal attributes, qualifications/experience, relationship with participants, assumptions, and/or presuppositions; potential or actual  interaction between researchers’ characteristics and the research  questions, approach, methods, results, and/or transferability | Not reported |
| Context | Setting/site and salient contextual factors; rationale | 8 |
| Sampling strategy | How and why research participants, documents, or events were selected; criteria for deciding when no further sampling was necessary (e.g., sampling saturation); rationale | 8-9 |
| Ethical issues pertaining to human subjects | Documentation of approval by an appropriate ethics review board and participant consent, or explanation for lack thereof; other confidentiality and data security issues | 12-13; 36 |
| Data collection methods | Types of data collected; details of data collection procedures including (as appropriate) start and stop dates of data collection and analysis, iterative process, triangulation of sources/methods, and modification of procedures in response to evolving study findings; rationale | 9 |
| Data collection instruments and technologies | Description of instruments (e.g., interview guides, questionnaires) and devices (e.g., audio recorders) used for data collection; if/how the instrument(s) changed over the course of the study | 9-11 |
| Units of study | Number and relevant characteristics of participants, documents, or events included in the study; level of participation (could be reported in results) | Not reported |
| Data processing | Methods for processing data prior to and during analysis, including transcription, data entry, data management and security, verification of data integrity, data coding, and anonymization/deidentification of excerpts | 10-12 |
| Data analysis | Process by which inferences, themes, etc., were identified and developed, including the researchers involved in data analysis; usually references a specific paradigm or approach; rationale | 10-13 |
| Techniques to enhance trustworthiness | Techniques to enhance trustworthiness and credibility of data analysis (e.g., member checking, audit trail, triangulation); rationaleb | 10 |
| Results/findings | | |
| Synthesis and interpretation | Main findings (e.g., interpretations, inferences, and themes); might  include development of a theory or model, or integration with prior  research or theory | 13-28 |
| Links to empirical data | Evidence (e.g., quotes, field notes, text excerpts, photographs) to substantiate analytic findings | 13-28 |
| Discussion | | |
| Integration with prior work, implications, transferability, and contribution(s) to the field | Short summary of main findings; explanation of how findings and conclusions connect to, support, elaborate on, or challenge conclusions of earlier scholarship; discussion of scope of application/ generalizability; identification of unique contribution(s) to scholarship in a discipline or field | 28-34; 35 |
| Limitations | Trustworthiness and limitations of findings | 31-34 |
| Other | | |
| Conflicts of interest | Potential sources of influence or perceived influence on study conduct and conclusions; how these were managed | 36 |
| Funding | Sources of funding and other support; role of funders in data collection, interpretation, and reporting | 37 |
