# Supplementary material for: Understanding the profile of community health workers in breast cancer screening education: women’s preferences and insights from a qualitative focus group study
Source: Int J Equity Health. 2025 Jul 4;24:193. doi: 10.1186/s12939-025-02508-0 (PMC12231706; doi:10.1186/s12939-025-02508-0)
Supplement: Supplementary file 3 — Supplementary Material 3 [file 12939_2025_2508_MOESM3_ESM.docx]

Final codebook manuscript: *"Understanding the profile of community health workers in breast cancer screening education: women's preferences and insights from a qualitative focus group study”.*

| **Main category** | **Subcategories/Axial codes**  *With corresponding descriptions* | **Open codes**  *With corresponding descriptions* |
| --- | --- | --- |
| **Gender of the CHW** | **A Male Person**  *This axial code encompasses all open codes indicating participants' preference for a male CHW.* | **Implicit reference to a male person**  *This code includes statements where participants implicitly refer to a male individual by using male pronouns such as "he" or "him" when discussing the CHW.* |
|  |  | **Explicit reference to a male person**  *In this code, participants explicitly refer to a male individual; instead of using pronouns such as "he" or "him," they use explicit terms to indicate a male person, such as "man," "sir," or by identifying a male volunteer.* |
|  |  | **Explicit preference for a male individual - Stereotypical beliefs** *In this code, participants explicitly refer to a male individual. This code reflects stereotypical beliefs or gender stereotypes. For example, it includes the notion that men inherently possess superior investigative skills or greater expertise in medical information due to their gender, or that a male individual is perceived as more serious or credible.* |
|  |  | **Explicit preference for a male individual - Male volunteer** *In this code, participants explicitly refer to a male individual. This code highlights the reasons why the male volunteer from FGD2 is perceived as a suitable CHW.* |
|  | **A female individual** *This axial code encompasses all open codes indicating that participants prefer a female CHW* | **Explicit reference to a female individual** *In this code, participants explicitly refer to a female individual. Instead of using pronouns such as "she" or "her," they use explicit terms to indicate a female person, such as "woman," "lady," "femme," or "helper."* |
|  |  | **Female pronouns** *In this code, participants implicitly refer to a female individual by using female pronouns such as "she" and "her."* |
|  |  | **Do not want a male individual** *In this code, participants clearly express that they do not wish to have a male individual as a CHW, for example, because they would feel uncomfortable with a man.* |
|  |  | **Explicit preference for a female individual - Religious beliefs** *In this code, participants describe the reasons why the immigrant community prefers a female CHW. It is important to note that these statements are made by women who are not part of this community.* |
|  |  | **Explicit preference for a female individual - With reason** *In this code, participants describe the reasons why they prefer a female individual as a CHW, such as the focus being on a female-oriented study, a female providing a greater sense of safety, or feeling more comfortable with a female.* |
|  | **No preference for gender** *This axial code includes all codes indicating that the participant(s) have no preference for the gender of the CHW.* |  |
| **Age of the CHW** | **Age Categories** *This axial code includes all codes that represent the different age categories/labels for the CHW.* | **20 – 30 years old** *In this code, participants describe their preference for someone between the ages of 20 and 30.* |
|  |  | **40 – 50 years old** *In this code, participants describe their preference for someone between the ages of 40 and 50.* |
|  |  | **50 – 60 years old**  *In this code, participants describe their preference for someone between the ages of 50 and 60.* |
|  |  | **Neither too young nor too old** *In this code, participants describe their preference for someone who is "neither too young nor too old," without specifying an exact age.* |
|  |  | **Same age as the target group** *In this code, participants describe their preference for someone of the same age as the target group, without specifying an exact age.* |
|  | **Age is unimportant** *This axial code indicates that the age of the CHW is considered unimportant.* |  |
|  | **Motivation for age** *This axial code includes all codes that provide reasons why participants prefer an older, younger, or a CHW from a specific age category.* | **Motivation for age - Experience** *In this code, participants describe that their motivation for the CHW's age is based on knowledge, particularly knowledge gained through experience. This experience is related to undergoing a mammogram.* |
|  |  | **Motivation for age - Communication** *In this code, one participant describes that their motivation for the CHW's age is based on communication.* |
|  |  | **Motivation for age - Patience** *In this code, participants describe that their motivation for the CHW's age is based on the belief that an older CHW would have more patience.* |
|  |  | **Motivation for age - Trustworthiness** *In this code, participants describe that their motivation for the CHW's age is based on the perception that an older CHW exudes greater trustworthiness.* |
|  |  | **Motivation for age – Time**  *In this code, participants describe that their motivation for the CHW's age is based on the belief that an older CHW would have more time available.* |
| **Network** | **Social network**  *This axial code encompasses all codes that highlight the importance of a social network.* | **A person from your family** *In this code, one participant expressed that they would appreciate it if the CHW were a member of their family.* |
|  |  | **A Person You Already Know**  *In this code, participants describe their preference for someone they already know or have previously met.* |
|  |  | **A person from the community** *In this code, participants describe the reasons why the CHW should come from an immigrant community to support individuals within that community. It is important to note that these statements were made by women who are not part of this community.* |
|  | **Professional network** *This axial code encompasses all codes that emphasize the importance of a professional network.* | **A person with connections to the hospital** *In this code, participants describe the reasons why it is important for the CHW to have connections to the hospital.* |
|  | **Cultural network** *This axial code encompasses all codes that highlight the importance of a cultural network.* | **A person who considers cultural differences** *This code highlights the importance of cultural competence, emphasizing the need for awareness of and respectful engagement with cultural diversity within a social network.* |
|  | **Health-related network** *This axial code encompasses all codes that emphasize the importance of a health-related network.* | **A person with experience related to (breast) cancer** *In this code, participants describe the reasons why it is important for the CHW to have connections with people who have experienced (breast) cancer. For example, the CHW might have a friend or family member with experience related to breast cancer, mammography, or both.* |
| **(Inter)personal traits and skills of the CHW** *This category emphasizes the importance of interaction between individuals, including communication, emotional expression, and the ability to effectively communicate and build relationships with others.* | **Communicative Traits** *This axial code encompasses all codes that reflect emotional traits that enable effective communication with the target group.* | **A polite person** *In this code, participants describe that the CHW should be a polite individual.* |
|  |  | **An unbiased person** *In this code, participants describe that the CHW should be someone with a neutral attitude, who does not judge and remains unbiased.* |
|  |  | **A person who Is communicatively flexible** *In this code, participants describe that the CHW should be someone who can adapt their level of literacy to the person they are addressing, ensuring that what they say is simple and clear. The CHW should adjust their manner of speaking (language use and choice of words) to suit the target audience.* |
|  | **Communicative skills** *This axial code includes all codes that demonstrate the skills a CHW requires for effective communication, such as language proficiency and digital communication abilities***.** | **A person proficient in Dutch** *This code indicates that participants believe the CHW should have proficiency in the Dutch language.* |
|  |  | **A person proficient in French** *This code indicates that participants believe the CHW should have proficiency in the French language.* |
|  |  | **A person proficient in Arabic** *This code indicates that participants believe the CHW should have proficiency in the Arabic language.* |
|  |  | **A person proficient in Ukrainian** *This code indicates that participants believe the CHW should have proficiency in the Ukrainian language.* |
|  |  | **A multilingual person** *This code indicates that participants believe the CHW should have proficiency in multiple languages.* |
|  | **Emotional Traits and Skills** *This axial code encompasses all codes that reflect emotional traits.* | **An understanding person** *This code indicates that participants believe the CHW should be an understanding individual.* |
|  |  | **An empathetic person** *This code indicates that participants believe the CHW should be an empathetic individual.* |
|  |  | **A warm person** *This code indicates that participants believe the CHW should be a warm individual.* |
|  |  | **A kind person** *This code indicates that participants believe the CHW should be a kind individual.* |
|  |  | **A friendly person** *This code indicates that participants believe the CHW should be a friendly individual.* |
|  |  | **A gentle person** *This code indicates that participants believe the CHW should be a gentle individual.* |
|  |  | **A social person** *This code indicates that participants believe the CHW should be a social individual.* |
|  |  | **A person who does not let others walk over them** *This code indicates that participants believe the CHW should be a strong individual who does not allow others to take advantage of them.* |
|  |  | **Resilient** *This code indicates that participants find it important for the CHW to have sufficient emotion-regulation skills to cope with their own experiences when these are shared.* |
|  | **Relational traits and skills** *This axial code encompasses all codes that describe traits and skills that help the CHW build strong, supportive relationships and create a safe and trusted environment for the target group.* | **A person who can take on a guiding role** *This code indicates that participants believe it is important for the CHW to be able to take on a guiding role.* |
|  |  | **A person who can listen** *This code indicates that participants believe it is important for the CHW to be someone who can listen well and be a "listening ear" for them.* |
|  |  | **A person who can motivate** *This code indicates that participants believe it is important for the CHW to be able to motivate them or take on a motivating role.* |
|  |  | **A person who can persuade** *This code indicates that participants believe it is important for the CHW to be able to persuade them or take on a persuasive role.* |
|  |  | **A person who is trusted** *This code encompasses all aspects related to trust. For example, when participants emphasize their preferences for a person with a certain profile, trust is a crucial factor, and/or the CHW will need to earn trust first or exude trustworthiness.* |
|  |  | **A person who makes others feel comfortable** *This code indicates that participants believe it is important for the CHW to be someone with whom they feel at ease and who can reassure them.* |
|  |  | **A social person** *This code indicates that participants believe the CHW should be a social individual.* |
|  |  | **A person who can adapt socially** *This open code includes all codes that indicate the importance of adapting to different situations. It refers to the ability to adjust to social situations, groups, and communities.* |
|  |  | **A person you can rely on** *This code indicates that participants believe it is important for the CHW to also take on a supportive role.* |
|  | **Physical appearance** *This axial code encompasses all codes that indicate whether or not physical appearance is considered important.* | **A person with a good appearance** *This code indicates that participants believe the CHW should have a good, "neutral" appearance***.** |
|  |  | **The CHW's appearance is not important** *This code indicates that participants believe it is not important how the CHW physically looks.* |
| **Professional kwalifications** | **Professional requirements** *This axial code encompasses the codes where participants mention that they would prefer the CHW to already practice or have practiced a (para)medical profession.* | **A retired doctor** *This code indicates that participants believe the CHW should be a retired individual with a medical degree or a former doctor’s assistant.* |
|  |  | **A person working as a nurse** *This code indicates that participants believe the CHW should be someone with a nursing degree or a background as a nursing assistant.* |
|  |  | **A person working as a general practitioner** *This code indicates that participants believe the CHW should be someone with a medical degree or experience as a doctor’s assistant.* |
|  |  | **A person working as a psychologist** *This code indicates that participants believe the CHW should be someone with a degree in psychology.* |
|  |  | **A professional** *This code indicates that participants believe the CHW can be a professional individual whose knowledge was acquired through an accredited higher medical education at a college or university. The specific field of study is not specified.* |
|  | **Educational level** *This axial code encompasses all codes where participants mention that they prefer the CHW to have completed education and/or additional training.* | **An educated person** *This code indicates that participants prefer a CHW who has completed education or additional training, specifically an accredited higher-level program.* |
|  |  | **A person who has received training** *This code indicates that participants prefer a CHW who has undergone additional training or education specific to the role. This refers to CHW training developed for the purpose of this study.* |
|  | **Knowledge** *This axial code encompasses all codes where participants specify the knowledge a CHW should possess.* | **Knowledge of breast cancer screening** *This code indicates that participants prefer a CHW who has knowledge about the subject matter, specifically mammography and/or breast cancer, including the steps involved in the process.* |
|  |  | **Knowledge beyond breast cancer screening** *This code indicates that participants prefer a CHW who possesses knowledge beyond the primary topic. This includes not only mammography and/or breast cancer but also the steps that follow after a diagnosis is made.* |
|  |  | **The right knowledge** *This code indicates that participants prefer a CHW who has the appropriate knowledge about the subject matter. The specific definition of "the right" knowledge is not specified.* |
| **Expertise Through Experience** | **Expert by experience** *This axial code describes all open codes indicating that the CHW must have experience with breast cancer, mammography, or both.* | **An expert by experience** *This code indicates that participants prefer a CHW who is an expert by experience. It is not specified whether this experience is related to breast cancer, mammography, or both.* |
|  | **(Breast) Cancer** *This axial code describes all open codes indicating that the CHW must have experience with breast cancer, mammography, or both.* | **Experience with (breast) cancer** *This code indicates that participants find it important for the CHW to be a current or former breast cancer patient or to have experience with cancer in general, although the specific type of cancer is not specified.* |
|  | **Mammography** *This axial code describes all open codes indicating that the CHW must have experience with breast cancer, mammography, or both.* | **Experience with mammography** *This code indicates that participants find it important for the CHW to have experience with undergoing a mammogram.* |
|  | **Life experience** *This axial code encompasses all references to the importance of the CHW having life experience.* | **Life experience** *This code indicates that participants find it important for the CHW to have life experience, a "backpack" of experiences.* |
|  | **No experience** | **No experience** *This code indicates that participants do not consider it important for the CHW to have prior experience with (breast) cancer and/or undergoing a mammogram.* |
